# A case of recurrent fixed drug eruption triggered by a hormonal intrauterine device

**DOI:** 10.1016/j.jdcr.2025.03.031

**Published:** 2025-04-16

**Authors:** Nardin Awad, Dominique Fontaine, Kristan Schiele, Aayushma Regmi, Aravindhan Sriharan, Natalie M. Fragoso

**Affiliations:** a Department of Dermatology, Dartmouth Hitchcock Medical Center, Lebanon, New Hampshire; b Department of Pathology, Dartmouth Hitchcock Medical Center, Lebanon, New Hampshire

**Keywords:** adverse drug reaction, autoimmune progesterone dermatitis, drug hypersensitivity, FDE, fixed drug eruption, intrauterine device, progesterone, rash

## Introduction

Fixed drug eruption (FDE) is a type IV hypersensitivity reaction and is the most common cutaneous adverse drug reaction. It typically presents as 1 or more round to oval well-demarcated erythematous macules, patches, or plaques. FDE classically occurs upon introduction to a drug and reoccurs in the same location upon subsequent exposures to the drug. Classic offending agents include antibiotics and nonsteroidal anti-inflammatory drugs; however, some rare and noteworthy agents have also been implicated that are not frequently recognized as potential triggers. Given the prevalence of intrauterine device (IUD) use, we report an important case of recurrent FDE caused by an IUD.

## Case

**Fig 1:**
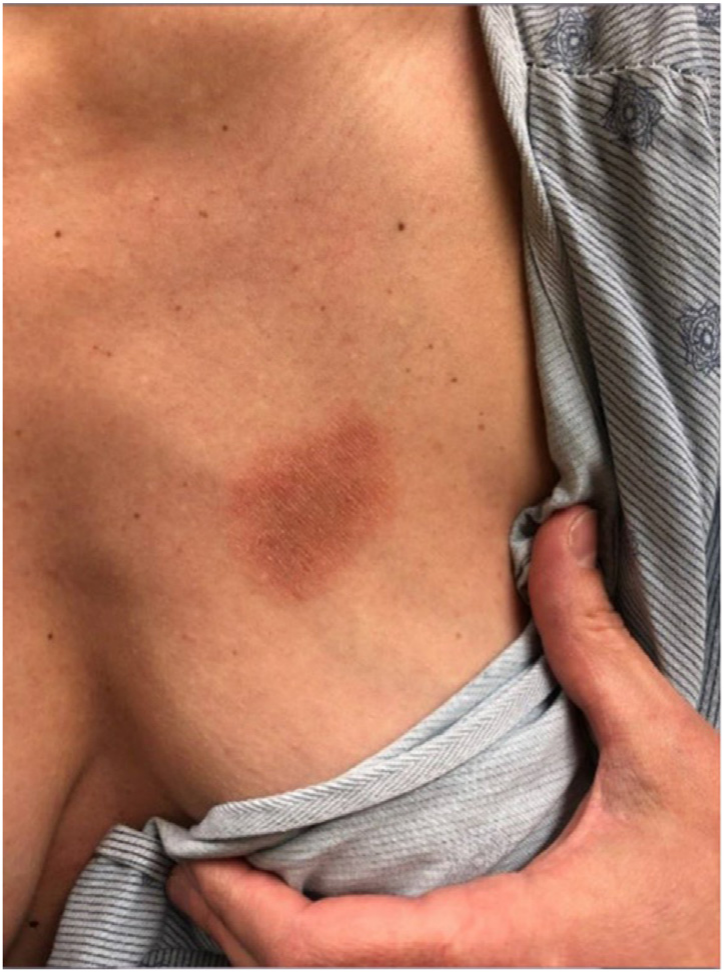
Fixed drug eruption. Erythematous thin plaque with central dusky hyperpigmentation on the left breast.

A 35-year-old female presented with a 6-month history of a rash on the left upper breast associated with intermittent pruritus (Fig 1). She had recently undergone a left breast ultrasound and mammography, which were negative. Medications included a levonorgestrel-releasing IUD, which was inserted approximately 8 weeks after giving birth and less than 4 weeks preceding the development of the rash. Notably, she reported a similar rash in the same location following hormonal IUD placement after a previous pregnancy, with resolution upon IUD removal. Physical examination revealed a 5 cm × 3.5 cm erythematous thin plaque with central dusky hyperpigmentation on the left breast.

**Fig 2:**
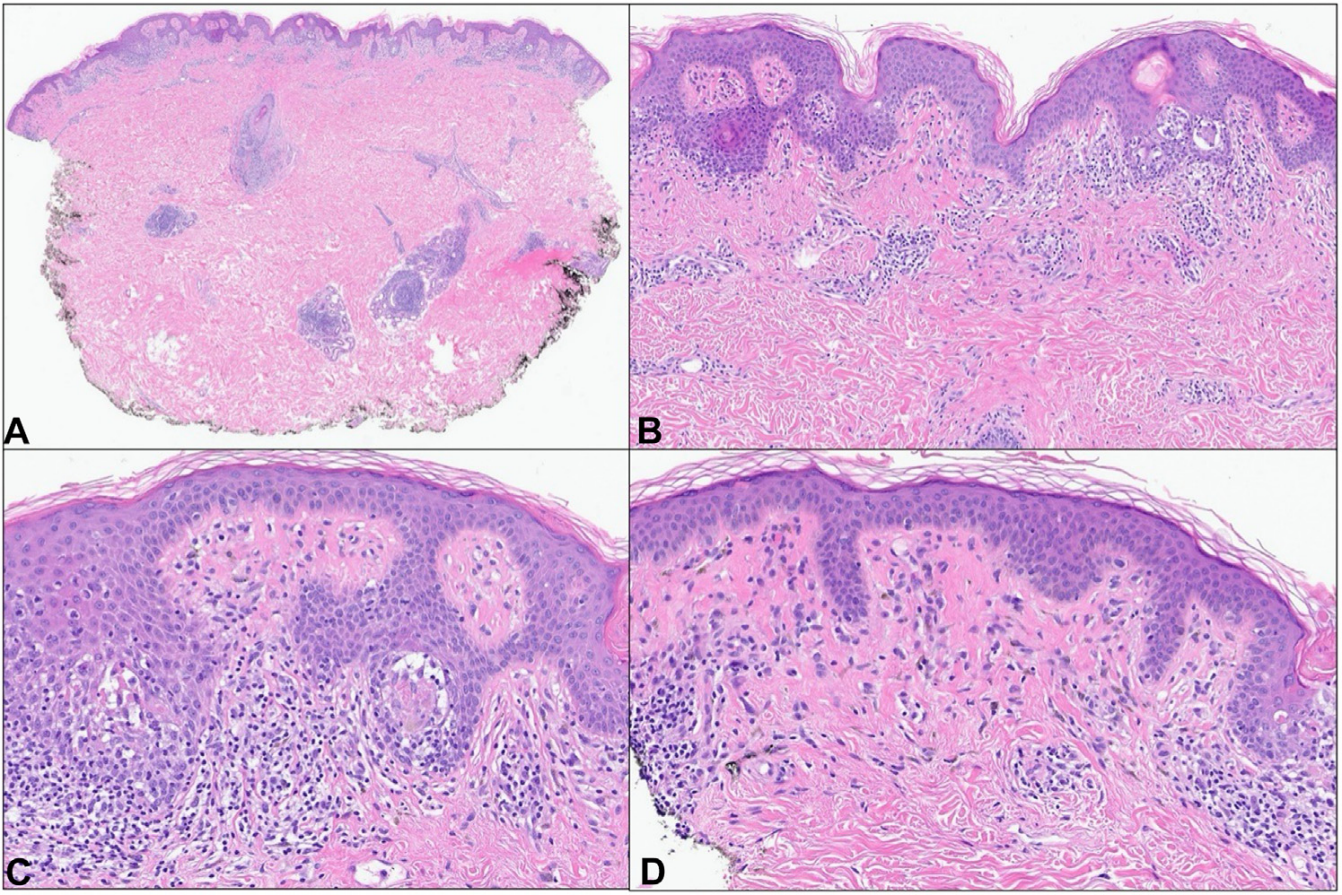
Hematoxylin and eosin staining revealing superficial and deep perivascular and periadnexal dermatitis with interface changes (**A**, 200×). High power revealed basket woven orthokeratosis and interface vacuolar degeneration with associated necrotic keratinocytes (**B**, 150×) (**C**, 200×). The superficial dermis showed mildly dense perivascular and interstitial lymphohistiocytic inflammation with scattered pigment-laden macrophages (**D**, 200×).

A punch biopsy was performed which demonstrated interface dermatitis with a superficial and deep perivascular and periadnexal lymphocytic infiltrate (Fig 2). Extensive tagging along the dermal-epidermal junction was noted, along with basal vacuolar degeneration and necrotic keratinocytes. Rare eosinophils were present. Autoimmune testing was positive for antinuclear and anti-Sjogren’s-syndrome-related antigen A/Ro antibodies, though lack of other symptoms or findings did not support tumid lupus. Following the replacement of the hormonal IUD with a copper IUD, the patient experienced complete resolution of her rash without any further recurrence.

## Discussion

Common triggers of FDE include nonsteroidal anti-inflammatory drugs, acetaminophen, and antibiotics. Over the past several years, many less typical drugs have been implicated, including antihistamines, oral hypoglycemic agents, and biologic drugs.^1^ To our knowledge, IUDs have not yet been reported as a cause of FDE. It is hypothesized that FDE occurs upon exposure to a specific drug which activates resident CD8+ T cells in the skin, leading to the release of inflammatory proteins and damage to the basal layer. Upon discontinuation of the inciting drug, inflammatory cells undergo apoptosis, allowing for basal layer regeneration. Interleukin-15 promotes CD8+ memory T cell production, which remains locally and may reactivate upon re-exposure to the drug, mounting a more rapid and severe immune response. The prolonged postinflammatory hyperpigmentation associated with FDE can partially be attributed to the melanin taken up by resident macrophages, which do not migrate after phagocytosis.

Autoimmune progesterone dermatitis (APD) was a differential diagnosis for our patient; however, APD is generally cyclical, associated with the luteal phase of the menstrual cycle, or occasionally triggered by pregnancy. In some patients, exogenous progesterone does trigger initial symptoms, but these patients then experience cyclic recurrence in association with the menstrual cycle following this initial sensitization.^2^ APD also frequently presents with other symptoms including angioedema, urticaria, anaphylaxis, or asthma.^3^ In contrast, our patient’s symptoms did not correlate with her menstrual cycle, were directly and solely associated with the presence and removal of her hormonal IUD on 2 distinct occasions, and did not include the aforementioned commonly concurrent symptoms of APD. A review of 89 patients with APD found that although symptom onset was found to be caused by a variety of triggers, all patients had recurring symptoms in relation to the menstrual cycle.^2^ There have been 2 previous reports of APD mimicking FDE. However, in both cases, symptoms recurred cyclically in conjunction with the menstrual cycle.^4^^,^^5^

The presence of antinuclear and anti-Sjogren’s-syndrome-related antigen A/Ro antibodies along with the perivascular and periadnexal lymphocytic infiltrate identified on histology prompted inclusion of tumid lupus erythematosus (TLE) in the differential diagnoses. Though often seronegative, TLE classically presents with photosensitive, nonscarring, annular, edematous plaques that demonstrate significant dermal mucin deposition and lymphocytic infiltrate without epidermal or junctional dermal-epidermal involvement. Histology from our case ruled out TLE, as it involved the dermal-epidermal junction and lacked dermal mucin deposition.^6^

There is a growing list of causative agents for FDE, and we hope this case report serves to shed light upon lesser-known agents. With an aging population, rising utilization of medications, and increasing development of new drugs, this list is likely to continue expanding. Although they may present infrequently, heightened awareness of atypical triggers of FDE will lead to more effective and timely patient management, ultimately improving patient outcomes.

## Conflicts of interest

Dr Fragoso has served as a consultant for UCB and as an investigator for Abbvie, Amgen, Acelyrin, Takeda, and Target Derm. Drs Awad, Fontaine, Schiele, Regmi, and Sriharan have no conflicts of interest to declare.

## References

[1] McClatchy J., Yap T., Nirenberg A., Scardamaglia L. (2022). Fixed drug eruptions – the common and novel culprits since 2000. J Dtsch Dermatol Ges.

[2] Nguyen T., Razzaque Ahmed A. (2016). Autoimmune progesterone dermatitis: update and insights. Autoimmun Rev.

[3] Foer D., Buchheit K.M., Gargiulo A.R., Lynch D.M., Castells M., Wickner P.G. (2016). Progestogen hypersensitivity in 24 cases: diagnosis, management, and proposed renaming and classification. J Allergy Clin Immunol Pract.

[4] Asai J., Katoh N., Nakano M., Wada M., Kishimoto S. (2009). Case of autoimmune progesterone dermatitis presenting as fixed drug eruption. J Dermatol.

[5] Mokhtari R., Sepaskhah M., Aslani F.S., Dastgheib L. (2017). Autoimmune progesterone dermatitis presenting as fixed drug eruption: a case report. Dermatol Online J.

[6] Saleh D., Grubbs H., Koritala T., Crane J.S. (2024).

